# Arsenic trioxide induces differentiation of cancer stem cells in hepatocellular carcinoma through inhibition of LIF/JAK1/STAT3 and NF‐kB signaling pathways synergistically

**DOI:** 10.1002/ctm2.335

**Published:** 2021-02-23

**Authors:** Xin Zhang, Bo Hu, Yun‐Fan Sun, Xiao‐Wu Huang, Jian‐Wen Cheng, Ao Huang, Hai‐Ying Zeng, Shuang‐Jian Qiu, Ya Cao, Jia Fan, Jian Zhou, Xin‐Rong Yang

**Affiliations:** ^1^ Department of Liver Surgery and Transplantation Liver Cancer Institute, Zhongshan Hospital Fudan University Shanghai China; ^2^ Key Laboratory of Carcinogenesis and Cancer Invasion (Fudan University), Ministry of Education Shanghai China; ^3^ Department of Pathology, Zhongshan Hospital Fudan University Shanghai China; ^4^ Cancer Research Institute Xiangya School of Medicine Central South University Changsha China; ^5^ Institutes of Biomedical Sciences Fudan University Shanghai China; ^6^ Shanghai Key Laboratory of Organ Transplantation Shanghai China; ^7^ State Key Laboratory of Genetic Engineering Fudan University Shanghai China

**Keywords:** arsenic trioxide, cancer stem cell, differentiation therapy, hepatocellular carcinoma

## Abstract

**Objective:**

Differentiation‐inducing therapy for tumors is a strategy that aims to induce the differentiation and maturation of cancer stem cells (CSCs). The differentiation‐inducing capacity of arsenic trioxide (ATO) in hepatocellular carcinoma (HCC) and the underlying mechanism were previously unknown.

**Methods:**

In the present study, we explored the ATO‐induced differentiation of CSCs in HCC by detecting the expression of CSC‐related markers and tumorigenicity variation in vivo and in vitro. We developed a combined chemotherapeutic approach to HCC by characterizing the effects of combinatorial treatment with 5‐fluorouracil (5‐FU)/cisplatin and ATO in vitro and in patient‐derived xenograft models. Changes in gene expression patterns were investigated by gene microarray analysis.

**Results:**

ATO effectively induced differentiation of CSCs by downregulation of CSC‐related genes and suppression of tumorigenicity capability. Combinatorial treatment with ATO and 5‐FU/cisplatin significantly enhanced therapeutic effects in HCC cells compared with the treatment with 5‐FU/cisplatin alone. Synergistic inhibition of the LIF/JAK1/STAT3 and NF‐kB signaling pathways by ATO and 5‐FU/cisplatin is a potential molecular mechanism underlying the differentiation effect.

**Conclusions:**

ATO induced the differentiation of HCC CSCs and potentiated the cytotoxic effects of 5‐FU/cisplatin through synergistic inhibition of the LIF/JAK1/STAT3 and NF‐kB signaling pathways. These results offer new insights for the clinical treatment of HCC.

## Graphical abstract

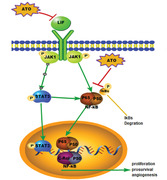

## 1 BACKGROUND

Liver cancer is the sixth most common cancer and the fourth most frequent cause of cancer‐related death globally.^1^ Hepatocellular carcinoma (HCC) accounts for about 90% of primary liver cancers and constitutes a major health problem.^2^ Surgery remains the most effective strategy with curative potential in well‐selected candidates; however, less than 30% of HCC patients are eligible for surgical intervention.^3^ For unresectable HCC, the prognosis is poor, and medical treatment is one of the few treatment choices for these patients.^4^ Conventional cytotoxic chemotherapy has marginal efficacy with frequent toxic effects because of primary chemoresistance and altered drug metabolism in HCC.^5^ The emergence of sorafenib represented a paradigm shift in the field of targeted HCC therapy; however, the survival benefit is limited, with low rates of tumor response.^6^, ^7^, ^8^ Meanwhile, lenvatinib was inferior to sorafenib except in overall survival for advanced HCC.^9^ Numerous clinical studies are currently underway to evaluate the efficacy of immune checkpoint inhibitors in many types of cancer, including HCC. Although the outcomes of these trials are highly anticipated, the latest results from a randomized Phase 3 study demonstrated that Opdivo (nivolumab) as a first‐line treatment in patients with unresectable HCC did not perform significantly better for its primary endpoint than sorafenib.^10^ Exploration of a novel effective medical treatment is essential to further improve the current management of HCC patients.

Cancer stem cells (CSCs) were identified as a small proportion of cancer cells that show stem‐like features, such as self‐renewal and indefinite proliferation.^11^, ^12^ Moreover, it has been recognized that CSCs show greater resistance to conventional chemotherapies than other cancer cell types.^12^ These features are believed to be the source of tumor recurrence and drug resistance. Therefore, therapies targeting these CSC subgroups are crucial to the prevention of tumor recurrence and reversion of drug resistance. Differentiation‐inducing therapy for tumors is defined as a therapeutic strategy that aims to induce reactivation of the endogenous differentiation program, as well as maturation of CSCs with loss of the tumor phenotype.^13^ Unlike the elimination of CSCs, which will inevitably cause damage to normal stem cells and affect the regeneration of normal tissue, inducing the differentiation of CSCs might be a safer and more promising strategy for cancer treatment.

Arsenic trioxide (ATO), a small‐molecule drug, was approved by the Food and Drug Administration (FDA) for leukemia treatment.^14^ ATO has been found to be effective against various hematologic malignancies and solid tumors.^15^, ^16^, ^17^, ^18^ However, our previous study in HCC indicated that the clinical effect of ATO varies across individuals, and high ATO concentrations are required to achieve therapeutic effects, which could lead to severe side effects.^19^ It is of paramount importance to determine how to alleviate the side effects of ATO, while maintaining its anti‐tumor efficacy, before its clinical application in HCC patients. Recent studies revealed that ATO can induce differentiation, and ATO treatment clears leukemia‐initiating cells in acute promyelocytic leukemia (PML) as well as in several types of solid tumors,^20^, ^21^, ^22^, ^23^ which might occur through induction of differentiation in CSCs. Meanwhile, the differentiation‐inducing capacity of ATO and its related molecular mechanism remain poorly understood in HCC.

Based on the above points, we explored the differentiation‐inducing capability of ATO administration in HCC cell lines in vivo and in vitro. We then developed a combined chemotherapeutic approach to HCC by characterizing the effects of combinatorial treatment with 5‐fluorouracil (5‐FU)/cisplatin and ATO in vitro and later confirming these results in HCC patient–derived xenograft models. Changes in gene expression patterns were further investigated by gene microarray analysis. We found that ATO induced differentiation of HCC CSCs and potentiated the cytotoxic effects of 5‐FU/cisplatin through synergistic inhibition of the LIF/JAK1/STAT3 and NF‐kB signaling pathways. These findings offer an alternative concept and practical approach in the management of HCC.

## 2 MATERIALS AND METHODS

### 2.1 Antibodies and reagents

CD133 microbeads and antibodies were purchased from Miltenyi Biotec (Bergisch Gladbach, Germany). Immunofluorescence antibodies against Nanog (cat no. 8750, rabbit polyclonal, 1:1000), ATP binding cassette subfamily G member 2 (ABCG2, cat no. 4477s, rabbit polyclonal, 1:1000), Sox2 (cat no. 67789, rabbit polyclonal, 1:1000), and Oct4 (cat no. 56159, rabbit polyclonal, 1:1000) were obtained from Cell Signaling Technology (Danvers, MA). Antibodies against JAK1 (cat no. 3332, rabbit polyclonal, 1:1000), STAT3 (cat no. 12640, rabbit monoclonal, 1:2000), phosphorylated STAT3 (cat no. 9145, rabbit monoclonal, 1:2000), and vimentin (cat no. 5741, rabbit monoclonal, 1:1000) were purchased from Cell Signaling Technology. Antibody specific for leukemia inhibitory factor (LIF, cat no. sc‐20087, rabbit polyclonal, 1:2000), α‐fetoprotein (AFP, cat no. sc‐8399, mouse monoclonal, 1:1000), glypican‐3(GPC3, cat no. sc‐390587, mouse monoclonal, 1:1000), phosphoenolpyruvate carboxykinase 1 (PCK1, cat no. sc‐271029 mouse monoclonal, 1:1000), and glucose‐6‐phosphate transporter (G6PT, cat no. sc‐293321, mouse monoclonal, 1:1000) was obtained from Santa Cruz Biotechnology (Santa Cruz, CA). Antibody against heppar‐1 was purchased from Dako Company (clone OCH1E5.2.10; 1:2000; Carpenteria, CA). Antibodies against E‐cadherin (cat no. ab1416, mouse monoclonal) and cytokeratin (cat no. ab215838, mouse monoclonal) were purchased from Abcam (Cambridge, UK). Antibodies against P50, P52, P65, c‐rel, RELB, and GAPDH were acquired from Cell Signaling Technology.

### 2.2 Cell culture

Huh7 and Hep3B human hepatocellular carcinoma cell lines were obtained from The Institutes of Biomedical Sciences, Fudan University (Shanghai, China). Cells were maintained in high‐glucose Dulbecco's modified Eagle medium (DMEM) or minimum essential medium (MEM) supplemented with 10% heat‐inactivated fetal bovine serum (FBS), 100 units/ml penicillin, and 100 mg/ml streptomycin at 37°C in a humidified atmosphere of 5% carbon dioxide. Primary HCC cells were identified and isolated from human HCC after surgery by directly cutting tumor fragments into tiny pieces of approximately 2 mm^3^. Cell suspensions were obtained after digestion with collagenase IV (Corning Life Sciences, Tewksbury, MA) for 2 h. Primary HCC cells were maintained in DMEM/F12 containing 10% heat‐inactivated FBS, 100 units/ml penicillin, and 100 mg/ml streptomycin at 37°C in a humidified atmosphere of 5% carbon dioxide. For primary cell cultivation, we obtained surgical specimens at the time of resection from all patients. All samples were received in the laboratory within 1 h, immediately mechanically disaggregated and digested with type IV collagenase (Gibco), and re‐suspended in DMEM medium. Single‐cell suspensions were obtained by filtration through a 40 μm filter. Red blood cells were lysed with ACK buffer (Invitrogen). The number of viable cells was counted and analyzed using Trypan blue. Isolated primary cells were then cultured in a serum‐free medium at a density of 20,000/well in an ultra‐low attachment six‐well plate. Primary cells within five passages were used for subsequent experiments.

**HIGHLIGHTS**

- ATO effectively induced differentiation of CSCs by downregulation of CSC‐related genes and suppression of tumorigenicity capability.
- ATO potentiated the cytotoxic effects of 5‐FU/cisplatin.
- ATO suppressed activities of SOD, CAT, and GSH and facilitated ROS accumulation.
- Synergistic inhibition of the LIF/JAK1/STAT3 and NF‐kB signaling pathways by ATO is a potential molecular mechanism underlying the differentiation effect.

### 2.3 Sphere formation assays

Our previous study confirmed that sphere‐forming cultures effectively enrich subpopulations of HCC cells with stem cell properties.^24^ Using this culture system, we further evaluated the ability of cells to form spheroids with or without pretreatment with ATO for 1 week by plating tumor cells (500–1000 cells per well) in a serum‐free chemically defined medium (CDM) on ultra‐low‐attachment six‐well plates (Corning Life Sciences). Spheroid formation was visually evaluated under a light microscope after 1 week at 37°C. Serum‐free CDM consisted of DMEM/F12 medium supplemented with 100 IU/ml penicillin, 100 ug/ml streptomycin, 20 ng/ml human recombinant epidermal growth factor (EGF), 20 ng/ml human recombinant basic fibroblast growth factor (bFGF), 1% nonessential amino acids, 1% GlutaMax, 2% B27 supplement (Invitrogen, Thermo Fisher Scientific, Waltham, MA), and 1% methylcellulose (Sigma‐Aldrich, St. Louis, MO).^25^

### 2.4 Flow cytometric analysis

Cells were labeled with phycoerythrin (PE)‐conjugated anti‐human CD133 antibody, and fluorescence data were acquired on a FACSCalibur flow cytometer (BD Biosciences, San Jose, CA). Isotype‐matched antibodies were used as controls. Briefly, 10 μl of antibody was added to the cell suspension. Samples were mixed thoroughly and incubated for 10 min in the dark at 2°C–8°C. Cells were washed by adding 1–2 ml buffer and centrifuged at 300*g* for 10 min. Supernatants were discarded and cell pellets were resuspended in a suitable amount of buffer for analysis by flow cytometry.

### 2.5 Immunofluorescence staining

Spheres‐forming cultures were fixed in 4% paraformaldehyde and blocked in 5% bovine serum albumin. Antibodies conjugated to PE were added and incubated overnight at 4°C. After washing with phosphate‐buffered saline (PBS) three times, nuclei of sphere cells were counterstained with DAPI (Sigma‐Aldrich). Images were captured using an IX‐71 fluorescent microscope (Olympus, Japan).

### 2.6 Magnetic labeling and separation

Cells were magnetically labeled with CD133 MicroBeads (Miltenyi Biotec) according to the manufacturer's instructions. The cell suspension was then loaded onto a positive selection MACS column, which was placed in the magnetic field of a MACS Separator. Flow‐through containing unlabeled cells was also collected. The column was removed from the separator and placed on an appropriate collection tube, and magnetically labeled cells were flushed out immediately.

### 2.7 Aldehyde dehydrogenase activity detection

Aldehyde dehydrogenase (ALDH) activity was assessed by flow cytometry with the use of an ALDEFLUOR kit (StemCell Technologies, France) in accordance with the manufacturer's instructions.

### 2.8 Animal experiments

CD133^+/–^ Huh7 cells were pretreated with the indicated concentration of ATO for 7 days and were then suspended in 150 μl serum‐free DMEM and Matrigel (1:1; BD Biosciences) before implantation subcutaneously into the right flanks of 6‐ to 8‐week‐old NOD/SCID male and female NOD/SCID mice. For the establishment of patient‐derived tumor xenograft (PDTX), we collected fresh surgical tumor samples with informed written consent from patients with HCC who had undergone hepatic resection at Zhongshan Hospital, Fudan University (Shanghai, China). The specimens were immediately preserved in ice‐cold serum‐free DMEM and sent to the laboratory within 2 h. Tumor samples were cut into small pieces of approximately 2 mm^3^, and four or five pieces of tissue were transplanted subcutaneously into the right flanks of 6‐ to 8‐week‐old NOD/SCID male and female mice NOD/SCID mice. Ethical approval was obtained from the Zhongshan Hospital Research Ethics Committee (approval no.: B2013‐021). Six mice were included for each group and indicated agents were intraperitoneally injected.

### 2.9 Cell proliferation assay, matrigel invasion, apoptosis assay, and immunohistochemistry

For the cell proliferation assay, cells with or without ATO pretreatment were aliquoted into a 96‐well plate at 3 × 10^3^ cells/100 μl per well in triplicate and incubated for 24 h. The medium was then replaced with complete medium containing 5‐FU/cisplatin. At indicated time points, 10 μl cell counting kit 8 (CCK‐8) solution (Dojindo, Rockville, MD) was added, and plates were incubated for another 2 h. The optical density (OD) at 450 nm was measured to determine the number of viable cells in each well. Cell viability was calculated using the following formula: cell viability (%) = (OD of the ATO treatment sample/OD of the control group) × 100%.

For the Matrigel invasion assay, 1 × 10^5^ Huh7 or Hep3B cells in 100 μl serum‐free DMEM were added to the well of an 8‐μm pore membrane Boyden chamber (Corning). The bottom chamber contained 10% FBS in DMEM as a chemoattractant. Cells were allowed to invade for 48 h; cells that had not penetrated the filters were then removed by scrubbing with cotton swabs. Chambers were fixed for 20 min at room temperature with 4% formaldehyde in PBS, stained in 0.1% crystal violet for 30 min, and rinsed in water. Cells that migrated to the bottom surface of the filter were considered to have invaded through the matrix and were counted under a light microscope. Assays were performed three times using triplicate wells.

Apoptosis was analyzed by flow cytometry using annexin V‐fluorescein isothiocyanate (FITC) or Annexin V‐PE Apoptosis Detection Kits (BD Biosciences) according to the manufacturer's protocol. Briefly, cells with or without ATO pretreatment were treated with 5‐FU or cisplatin for 48 h, then harvested and suspended in binding buffer (1×). An aliquot of 100 μl was incubated with 5 μl annexin V‐FITC/PE and 5 μl propidium iodide or 7‐aminoactinomycin D for 15 min in the dark, and 400 μl binding buffer was added to each sample. The stained cells were analyzed by flow cytometry within 1 h.

For immunohistochemistry, slides were dewaxed by heating at 60°C overnight and washing twice, 10 min each, in xylene. The tissues were rehydrated in a graded ethanol series of 5‐min washes in 95%, 80%, 75% ethanol, and distilled water. Endogenous peroxidase activity was blocked by incubation in 3% hydrogen peroxide for 15 min. Antigen retrieval was performed by heating the samples at 95°C for 15 min in 10 mM sodium citrate (pH 6.0). After blocking with the universal blocking serum for 60 min, the samples were incubated with primary antibodies at 4°C overnight. The sections were then incubated with biotin‐labeled secondary antibody and streptavidin‐peroxidase for 30 min each. The samples were developed using 3, 3′‐diaminobenzidine and counterstained with hematoxylin. The sections were then dehydrated following a standard procedure and sealed with coverslips.

### 2.10 Reverse transcription‐quantitative PCR and western blot analysis

Reverse transcription‐quantitative PCR (RT‐qPCR) and western blot analysis were performed as previously described.^19^ Total RNA was extracted from HCC cell lines using Trizol reagent (Invitrogen) according to the manufacturer's instructions. Relative mRNA expression was measured by RT‐qPCR using the Mastercycler ep realplex (Eppendorf, Hamburg, Germany). An SYBR PrimeScript RT‐PCR Kit (Takara, Japan) was used according to the manufacturer's instructions. GAPDH was used as an internal control. The primers are listed in Table S1.

Relative mRNA levels were calculated based on the cycle threshold (*Ct*) values, normalized to GAPDH expression level, according to the following equation: 2^–Δ^ *^Ct^* [ΔCt = *Ct* (target) – *Ct* (GAPDH)]. All experiments were performed in triplicate.

For western blot analysis, total protein was extracted in lysis buffer for 30 min on ice. Equal amounts of protein were separated by 8% sodium dodecyl sulfate‐polyacrylamide gel electrophoresis and electrophoretically transferred to polyvinylidene difluoride membranes (Millipore, Billerica, MA) using a mini trans‐blot apparatus (Bio‐Rad Laboratories, Hercules, CA). Membranes were blocked with Tris‐buffered saline with Tween (TBST) containing 5% nonfat dry milk for 1 h and incubated with primary antibody overnight at 4°C. Membranes were then washed three times with TBST and incubated with horseradish peroxidase‐conjugated immunoglobulin G (Chemicon, Temecula, CA) at a 1:5000 dilution for 1 h at room temperature. Blots were developed using a Pierce enhanced chemiluminescence kit (Thermo Fisher Scientific). Each experiment was repeated at least three times.

### 2.11 Microarray‐based gene expression profile

The Human Genome U133 Plus 2.0 microarrays used to profile changes between ATO‐treated and DMSO‐treated Huh7 and Hep3B cells were constructed by Affymetrix (Santa Clara, CA). Arrays were scanned using the GeneChip Scanner 3000 (Cat#00‐ 00212, Affymetrix) and Command Console Software 3.1 (Affymetrix) with default settings. Raw gene expression data were preprocessed using the Robust Multi‐array average algorithm, log‐transformed, and quartile normalized (implemented with R package “simpleaffy”). The similarity between two samples was calculated by Spearman's rank correlation coefficient, and the association between gene expression and sorafenib sensitivity was identified by a Student's *t*‐test (*p* < 0.01). The functional enrichment analyses of differentially expressed genes were performed using the Qiagen Ingenuity Pathway Analysis (IPA) software (www.qiagen.com/ingenuity). The complete dataset is available as GEO (Gene Expression Omnibus) profiles on the GEO database (www.ncbi.nih.gov/ geo/; GEO accession number GSE128517).

### 2.12 Plasmid construction and transfection

Plasmids were constructed by restriction‐enzyme double digestion and ligation. pcDNA‐JAK1 and pcDNA‐STAT3 were based on the pcDNA backbone with insertion of the coding region for JAK1 and STAT3, respectively. The sequences of P65 and STAT3 siRNA were listed in Table S2. Plasmid transfection was performed with Lipofectamine 2000 (Invitrogen) according to the supplier's protocol.

### 2.13 Measurement of reactive oxygen species

Reactive oxygen species (ROS) were measured using an oxidation‐sensitive fluorescent probe 2′,7′‐dichlorofluorescin diacetate (DCFH‐DA; Beyotime Institute of Biotech, China), following the manufacturer's instruction. Cells were incubated with DCFH‐DA (diluted in serum‐free DMEM to the concentration of 10 mM) for 30 min at 37℃ in the dark. The reaction mixture was discarded and cells were washed by serum‐free DMEM twice. Intracellular fluorescence was observed using an upright fluorescence microscope immediately. For flow cytometry analysis, cells were resuspended in serum‐free DMEM containing DCFH‐DA (10 mM) and incubated for 30 min at 37℃ in the dark. After washed by serum‐free DMEM twice, cells were resuspended in 350μl PBS for cytometry analysis.

### 2.14 Measurement of superoxide dismutase, catalase, and glutathione

The activities of superoxide dismutase (SOD), catalase (CAT), and glutathione (GSH) were all measured using assay kits according to manufacturer's instructions (Beyotime Institute of Biotech, China). More details could be found in a previously published article.^26^

### 2.15 Luciferase reporter assays

The NF‐kB promoter was amplified and subcloned into pGL 3.0 luciferase reporter plasmid. Cells were seeded into 96‐well plates and treated with ATO, pcDNA‐JAK1, and pcDNA‐STAT3 for 24 h, then transfected with pRL‐CMV *Renilla* luciferase reporter and the pGL 3.0 luciferase reporter plasmid for 48 h. Firefly and *Renilla* luciferase activities were measured using a dual‐luciferase reporter system.

### 2.16 Statistical analysis

Statistical analyses were performed with SPSS version 20.0 for Windows (IBM, Armonk, NY) and GraphPad Prism (version 7.0) for Windows (GraphPad Software, San Diego, CA). Student's *t*‐tests were used for comparison between groups. All tests were two‐tailed and *p *< 0.05 was considered statistically significant.

## 3 RESULTS

### 3.1 ATO altered tumor cell behavior and reduced CSC biomarker expression but had no effect on apoptosis

**FIGURE 1:**
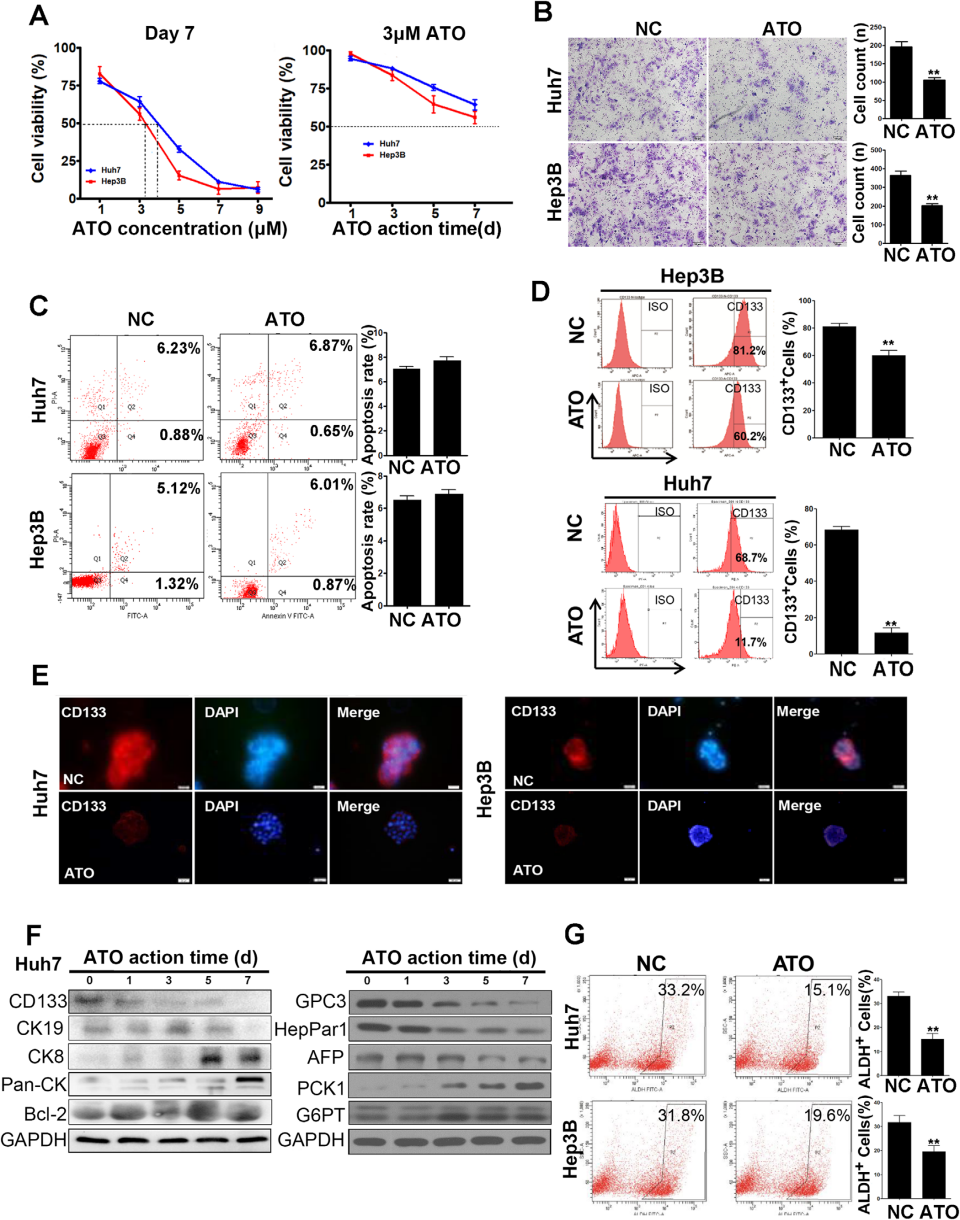
Low‐dose ATO altered tumor‐cell behavior and reduced CSC biomarker expression. (A) CCK‐8 tests were performed to determine the 7‐day IC_50_ of ATO in Huh7 and Hep3B cells. Cells were treated with the indicated concentrations of ATO for 7 days (left panel). Note that 3 μM ATO was used for subsequent CCK‐8 test (right panel). (B) Transwell assays indicated that cells treated with 3 μM ATO were less invasive than the control group. (C) Apoptosis was detected by annexin V‐FITC staining. Compared to the control, 3 μM ATO did not effectively trigger cell apoptosis. (D) Huh7 or Hep3B cells were pretreated with ATO, and the CD133^+^ subpopulation was analyzed by flow cytometry. (E) Immunofluorescence staining of CD133 in Huh7 and Hep3B cell spheres with or without ATO treatment (magnification: 200×). (F) Western blot analysis showed changes in levels of HCC CSC markers CD133, CK19, epithelial marker CK8, Pan‐CK, and apoptosis‐related protein BCL‐2 with ATO therapy for 9 days. (G) ALDH activity was analyzed by flow cytometry in CD133^+^ Huh7 and Hep3B cells and represented the proportion of ALDH^+^ cells, which was largely reduced by ATO therapy

As differentiation therapy is recognized as a time‐dependent process and does not aim to eliminate cancer cells but to induce the maturation of CSCs with loss of the tumor phenotype, we performed 7‐day IC_50_ tests in both cell lines to determine the appropriate dosage of ATO. In Huh7 and Hep3B cells, the 7‐day IC_50_ of ATO was 3.78 and 3.25 μM, respectively (Figure 1A, left). We then used 3 μM ATO for subsequent tests. With this dosage, ATO slightly inhibited cell growth, and the viability of tumor cells remained greater than 50% after 7 days in both cell lines (Figure 1A, right). Matrigel invasion assays showed that the number of invasive cells decreased significantly (relative cell counts of 101.2 ± 11.4 and 203.6 ± 7.1 for Huh7 and Hep3B, respectively) compared with the control group (relative cell counts of 190.6 ± 13.4 and 364.5 ± 23.4 for Huh7 and Hep3B, respectively), after exposure to 3 μM ATO for 7 days (*p *< 0.05) (Figure 1B). However, 3 μM ATO did not significantly promote apoptosis in this therapeutic strategy (Huh7, NC vs. ATO: 7.06 ± 0.17% vs. 7.74 ± 0.29%, *p *= 0.12; Hep3B, NC vs. ATO: 6.47 ± 0.19% vs. 6.91 ± 0.18%, *p *= 0.15) (Figure 1C).

Furthermore, we found that the proportion of CD133^+^ tumor cells significantly decreased from 68.41 ± 1.83% to 11.63 ± 2.71% (*p *< 0.001) in Huh7 cells and from 81.20 ± 2.14% to 60.17 ± 3.61% in Hep3B cells (*p *= 0.007) after the treatment for 7 days (Figure 1D).

Nonadherent tumor spheres under serum‐free conditions efficiently enriched cancer stem cells in vivo.^27^ After Huh7 and Hep3B cells formed tumor spheres, we found that the fluorescence intensity of CD133 in tumor spheres was significantly enhanced and less fluorescence intensity was shown when tumor cells were pretreated with ATO (Figure 1E). Western blot analysis indicated the protein levels of stem cell marker CD133 were reduced and reached their lowest levels on day 7. HCC‐related proteins such as CK19, Glypican‐3 (GPC3), HepPar1, and α‐fetoprotein (AFP) displayed a time‐dependent downregulation by ATO. However, expression of epithelial markers CK8, cytokeratin, and two liver‐specific proteins (PCK1 and G6PT) was enhanced. The level of BCL‐2, an apoptosis‐related protein, did not change with ATO administration, which is consistent with the results of our previous apoptosis assay (Figure 1F). As another CSC marker, ALDH activity was analyzed by flow cytometry in Huh7 and Hep3B cells. ALDH activity represented the proportion of ALDH^+^ cells, which was reduced from 33.10 ± 1.70% to 15.27 ± 2.28% in Huh7 (*p *= 0.003) and from 31.80 ± 2.83% to 19.57 ± 2.45% in Hep3B (*p *= 0.03) cells after ATO treatment (Figure 1G).

### 3.2 ATO downregulated expression of stemness‐related genes and inhibited self‐renewal and tumorigenic capacity of HCC CSCs

**FIGURE 2:**
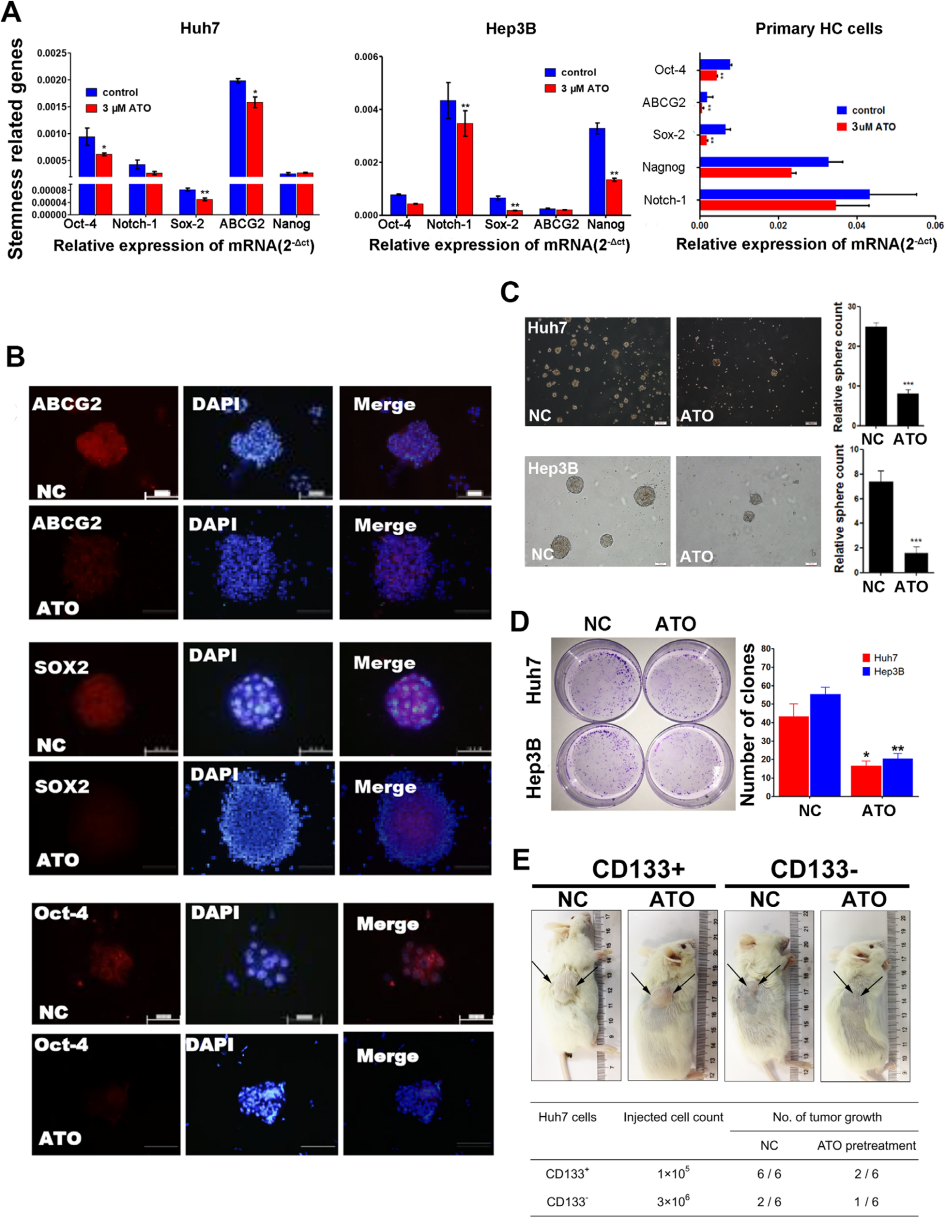
Low‐dose ATO downregulated expression of stemness‐related genes and inhibited self‐renewal and tumorigenic capacity of HCC CSCs. (A) Relative mRNA expression of stemness‐related genes *OCT4, NOTCH1, SOX2, ABCG2*, and *NANOG* altered by ATO treatment in Huh7, Hep3B, and primary HCC cells. (B) Immunofluorescent staining of stemness‐related proteins ABCG2, SOX2, OCT4, and CD133 in Huh7 cell spheres with or without ATO treatment (magnification: 200×). (C) ATO impaired sphere‐forming ability of Huh7 and Hep3B cells (magnification: 200×). (D) Upon exposure to ATO, the number of clones diminished dramatically in Huh7 cells and Hep3B cells. (E) NOD/SCID mice were subcutaneously inoculated with CD133^+^ or CD133^‐^ Huh7 cells to explore the tumorigenic potential altered by ATO

We further examined the expression of several stemness‐related genes by RT‐qPCR analysis during the process of differentiation therapy. Compared to the control, *OCT4*, *SOX2*, and *ABCG2* mRNA expression was significantly decreased in Huh7 cells pretreated with ATO. In Hep3B cells, *NOTCH1, SOX2*, and *NANOG* mRNA expression was significantly reduced in the ATO group. Meanwhile, expression of *OCT4*, *SOX2*, and *ABCG2* mRNA was significantly decreased in primary HCC cells after ATO pretreatment (Figure 2A). Immunofluorescent staining of Huh7 spheres also confirmed the reduced intensity of fluorescence staining of OCT4, SOX2, and ABCG2 in the ATO group (Figure 2B).

We further compared the number of spheres in cultures treated with or without ATO treatment. The number of spheres was significantly decreased from 25.12 ± 1.08 to 8.20 ± 1.21 in Huh7 cells (*p *< 0.001), and from 7.41 ± 0.87 to 1.62 ± 0.51 in Hep3B cells (*p* < 0.001) (Figure 2C). Similar results were observed in the plate clone formation assay, and the number of clones dropped dramatically from 43.31 ± 6.82 to 16.73 ± 2.41 in Huh7 cells (*p *= 0.02) and from 55.34 ± 3.76 to 20.35 ± 2.96 in Hep3B cells (*p *< 0.001) upon exposure to ATO (Figure 2D).

We inoculated NOD/SCID mice subcutaneously with 1 × 10^5^ CD133^+^ Huh7 cells or 3 × 10^6^ CD133^–^ Huh7 cells to compare the tumorigenic potential of ATO‐pretreated and untreated HCC cells. ATO pretreatment successfully inhibited tumor‐forming capacity of CD133^+^ Huh7 cells in vivo (Figure 2E). Compared to the NC group, only two of the six mice had visible tumors. As expected, although many more CD133^–^ Huh7 cells were inoculated, the visible tumor formation rate was low in both treatment and control groups.

### 3.3 ATO enhances the sensitivity of HCC CSCs to chemotherapeutic drugs in vitro and in vivo

**FIGURE 3:**
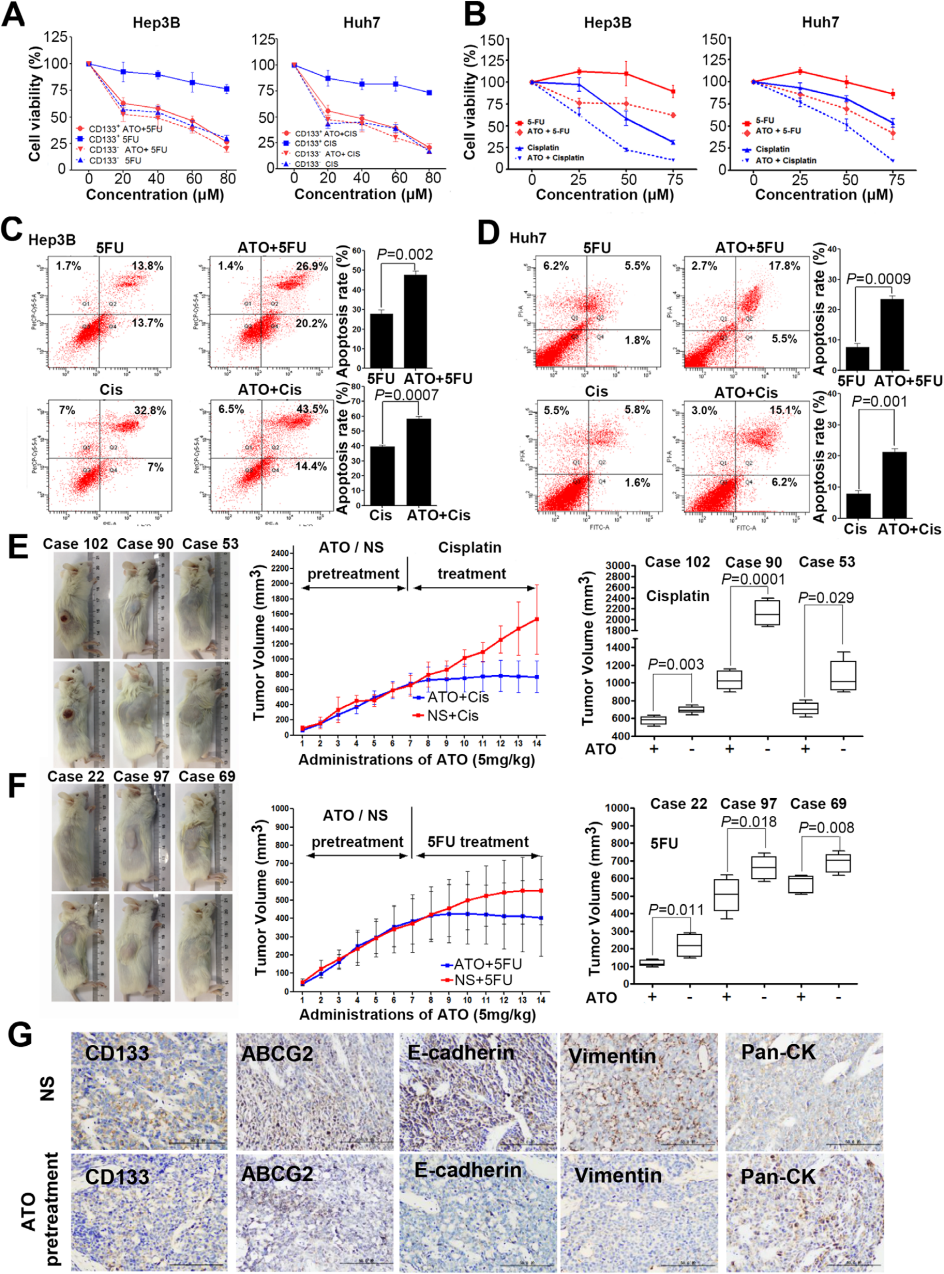
The combined ATO and 5‐FU/cisplatin treatment effectively suppresses tumor growth and promotes apoptosis of HCC cells in vivo and in vitro. (A and B) Effects of different treatments on growth of CD133^+/−^ Huh7 and Hep3B cells. (C and D) Apoptosis of Hep3B and Huh7 cells receiving the indicated treatments was evaluated by annexin V‐FITC/PE staining. The left panel depicts the proportion of apoptotic cells, and the right panel shows the quantitative results. (E) PDTX models were given the indicated treatments. The left panel shows pictures taken from one of the six mice of each group and shows the tumor volume after 14 doses. The middle panel indicates dynamic tumor growth curves. The right panel depicts the statistical analysis of tumor volume for each case receiving indicated treatments. (F) Representative images of tumor specimens analyzed using immunohistochemistry (magnification: 200×)

Since we assumed that the differentiation‐inducing capability of ATO would enhance the sensitivity of HCC CSCs to chemotherapeutic drugs, we tested this hypothesis by examining the effect of combinatorial treatment of Huh7 and Hep3B cells with ATO and 5‐FU/cisplatin. Using a MACS cell separation system, CD133^+^ and CD133^–^ cells were isolated and used in the following cell proliferation assay. As shown in Figure 3A, the inhibition of cell growth by combinatorial treatment with ATO and 5‐FU or cisplatin was more pronounced in CD133^+^ Huh7 and Hep3B cells than in CD133^–^ cells. In addition, for the whole population, the combinatorial treatment had a stronger inhibitory effect on cell growth in both cell lines (Figure 3B). Moreover, the combinatorial treatment triggered a higher level of apoptosis in both cell lines. In Hep3B cells, the apoptosis rate was 47.42 ± 1.84% in the ATO–5‐FU group versus 27.81 ± 2.11% in the 5‐FU group (*p *= 0.002) and 56.26 ± 2.27% in the ATO‐cisplatin group versus 39.21 ± 1.03% in the cisplatin group (*p *< 0.001) (Figure 3C). In Huh7 cells, the apoptosis rate was 23.73 ± 2.04% in the ATO–5‐FU group vs. 8.16% ± 1.57 in the 5‐FU group (*p *< 0.001) and 21.34 ± 2.23% in the ATO‐cisplatin group vs. 8.42 ± 1.68% in the cisplatin group (*p *= 0.001) (Figure 3D). The above in vitro results prompted us to further investigate the combinatorial use of ATO and 5‐FU/cisplatin in vivo. We established patient‐derived tumor xenograft (PDTX) models based on six HCC cases. Patient characteristics are summarized in Table 1. For each case, six mice were included and randomly divided into two groups. The experimental group was pretreated with ATO and the control group was given normal saline (NS) daily. After seven doses, both groups were treated with cisplatin (10 mg/kg) or 5‐FU (10 mg/kg). Compared to control, ATO treatment alone had little impact on tumor growth. However, after seven doses of ATO pretreatment, dramatic inhibition of tumor growth occurred with cisplatin/5‐FU treatment. After a total of 14 doses, significant differences were observed in tumor size between the ATO pretreatment group and the NS control group in each case (Figure 3E and F). Subsequent immunohistochemical staining of tumors showed downregulation of CD133, ABCG2, E‐cadherin, and vimentin, whereas cytokeratin expression was significantly upregulated in the ATO pretreatment group (Figure 3G). Taken together, these results suggest that ATO induced differentiation of HCC CSCs, eliminating HCC CSC chemoresistance and enhancing the cytotoxicity of 5‐FU/cisplatin.

**TABLE 1:** Clinical and pathologic features of patient material established animal models of HCC PDX

Patient no.	Age/sex	Patholoic diagnosis	TNM stage	AFP level (ng/ml)	Tumor size (cm)	Tumor number	Vascular invasion	Edmonson grade
102	64/M	HCC	T2N0M0	2.6	4 × 3.5 × 3	1	No	II
90	46/M	HCC	T2N0M0	5821	4.5 × 4 × 4	1	No	II
53	53/M	HCC	T1N0M0	60,500	8 × 8 × 7	1	Yes	II
22	39/M	HCC	T2N1M0	76	1.5 × 1 × 1	1	No	II
97	34/M	HCC	T2N0M0	18.3	6.5 × 5 × 5	1	Yes	II
69	61/F	HCC	T3aN0M0	51,144	7 × 4 × 4	2	Yes	II

Abbreviations: AFP, alpha‐fetoprotein; HCC, hepatocellular carcinoma; PDX, patient derived xenograft.

### 3.4 ATO induces inhibition of the JAK‐STAT signaling pathway in HCC

**FIGURE 4:**
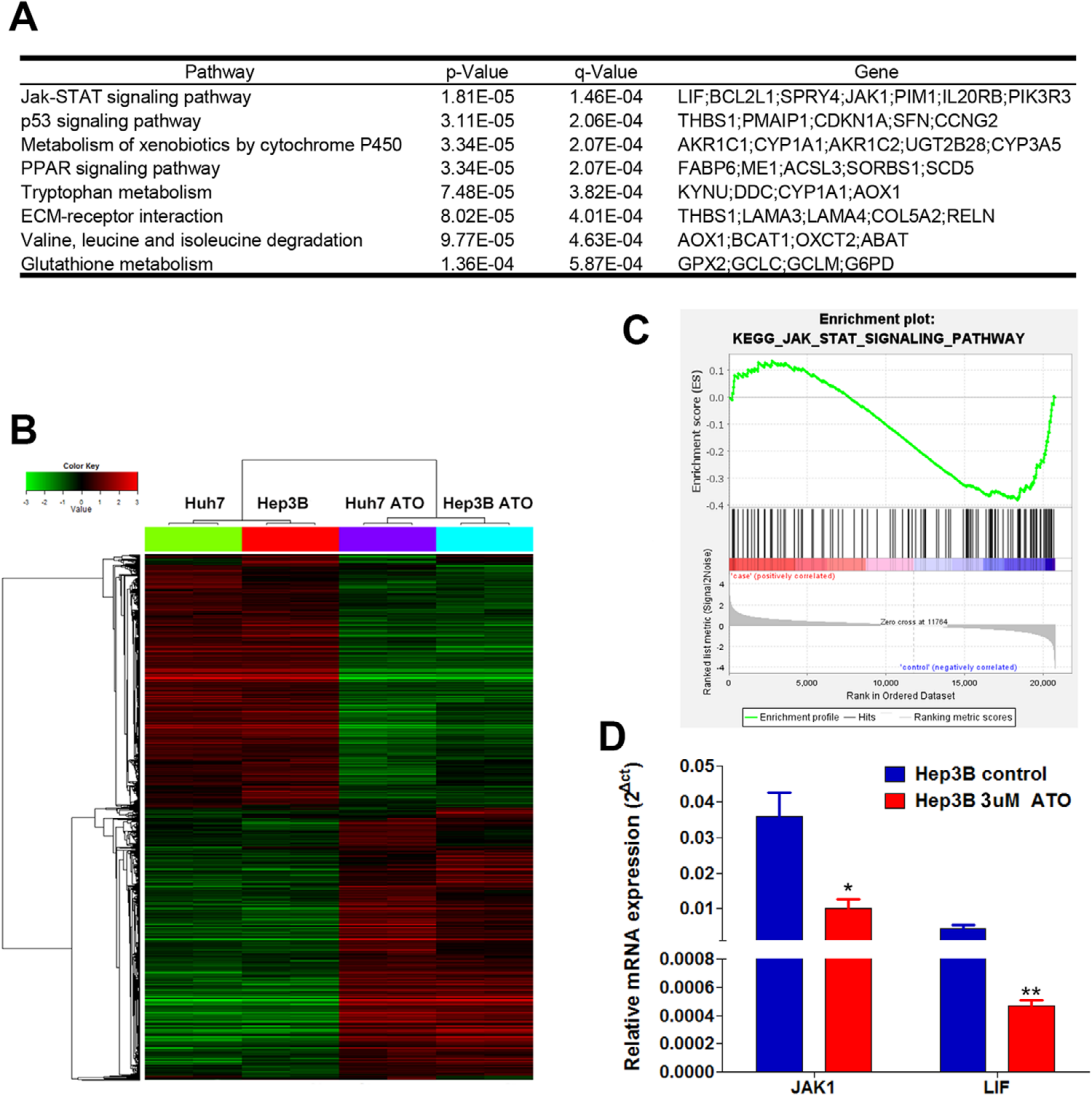
Gene microarray analysis followed by KEGG pathway analysis (A) The top eight ranked up‐or downregulated genes that underwent bioinformatic analysis using the KEGG are shown. (B) Supervised cluster analysis depicted two different gene expression patterns between two groups. (C) The gene set enrichment analysis was introduced to provide the enrichment plot of the JAK‐STAT signaling pathway, which indicated that most of the genes are downregulated in the analyzed pathway. (D) Low‐dose ATO significantly downregulated relative *JAK1* and *LIF* mRNA expression (*JAK1*, *p < 0.05 vs. control; *LIF*, **p < 0.01 vs. control, mean ± SEM, *t*‐test)

To further explore the mechanism of ATO‐induced differentiation, we performed gene microarray analysis to compare the differential gene expression profiles of ATO‐treated (3 μM ATO treatment for 7 days) and negative control cells; 422 common differentially expressed genes were found between the ATO‐treated and negative control group in Huh7 and Hep3B cell lines. Of these, 234 genes were upregulated (ratio > 1.50) and 188 genes were downregulated (ratio < 0.67). Those differentially expressed genes were then enrolled to the bioinformatics analysis to calculate the enriched Kyoto Encyclopedia of Genes and Genomes (KEGG) pathways (Figure 4A). Supervised cluster analysis depicted two different gene expression patterns between two groups regardless of the cell line (Figure 4B). Numerous pathways, including JAK‐STAT,^28^ p53,^29^ PPAR,^30^ and GSH metabolism,^31^ have been reported in stem cell regulation, tumor inhibition, and control of cell growth and differentiation. We further conduct the gene set enrichment analysis (GSEA) to calculate the enrichment score and normalized enrichment score of these pathways (Table S3). Of these, the JAK‐STAT signaling pathway drew our attention as it ranked first among pathways and was closely related to stem cell maintenance and regulation.^28^ The enrichment plot of the JAK‐STAT signaling pathway using GSEA was provided (Figure 4C). We mainly focused on the LIF‐JAK‐STAT axis. ATO significantly downregulated relative *JAK1* and *LIF* mRNA expression, respectively, from 0.036 ± 0.006 to 0.010 ± 0.003 (*p *= 0.021) and 0.004 ± 0.0008 to 0.0005 ± 0.00003 (*p *= 0.007). In addition, the RT‐qPCR results correspond well to the microarray data (Figure 4D).

### 3.5 ATO synergistically depresses the LIF/JAK/STAT and NF‐kB pathways

**FIGURE 5:**
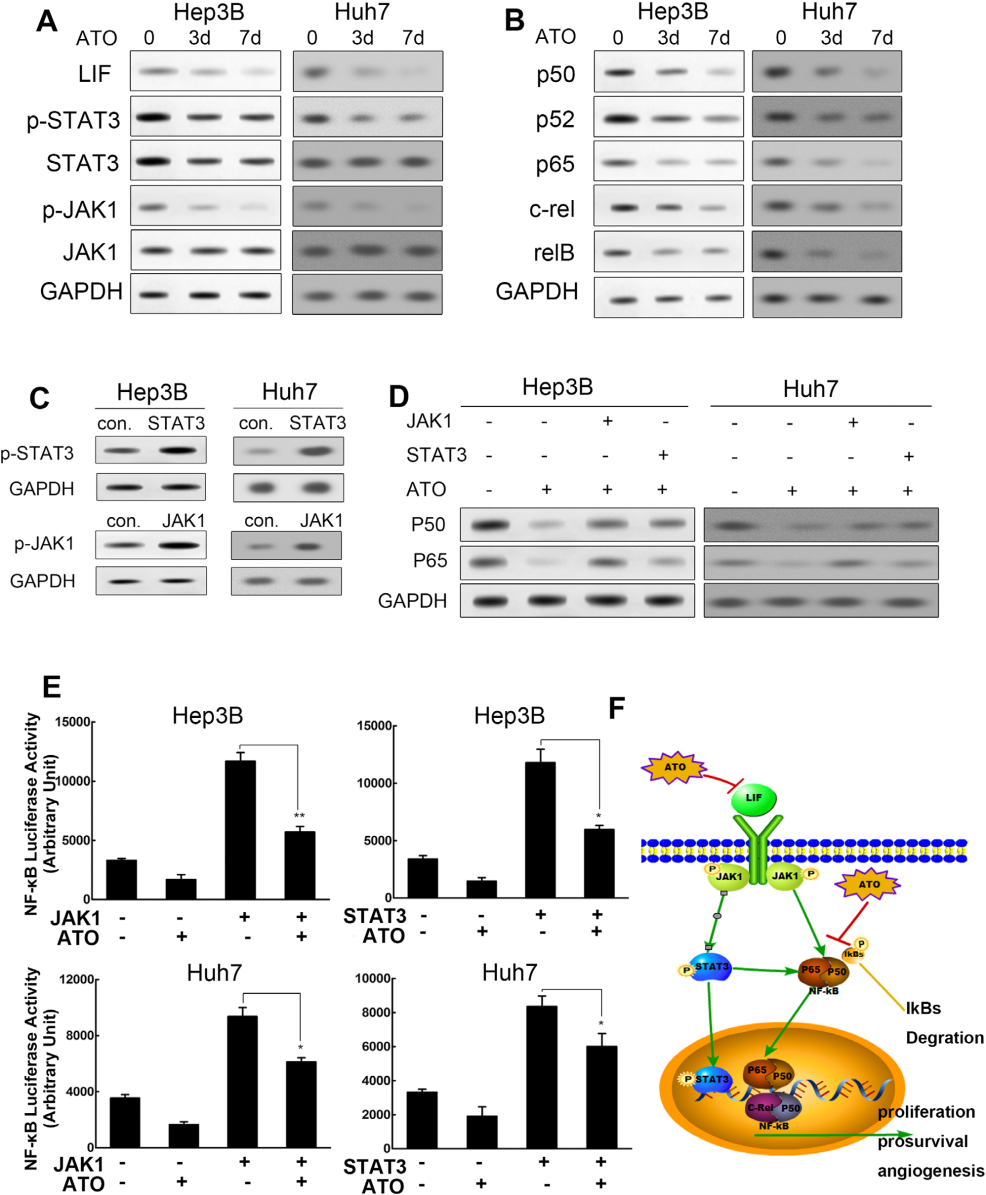
ATO synergistically depresses LIF/JAK/STAT and NF‐kB pathways (A) Cells receiving the indicated treatments were harvested for western blot analysis and probed for LIF, JAK1, STAT3, phosphorylated JAK1, and phosphorylated STAT3. (B) Cells receiving the indicated treatments were harvested for western blot analysis and probed for five members of the NF‐kB family—P50, P52, P65, c‐rel, and RELB. (C) The overexpression efficiency of JAK1 and STAT3 was detected by western blot analysis in both cell lines. (D) Western blot analysis depicted protein level variation of P50 and P65 under indicated management. (E) Luciferase activity of NF‐kB under indicated treatment in Huh7 (*p < 0.05 vs. pcDNA‐JAK1 or pcDNA‐STAT3 alone treatment, mean ± SEM, *t*‐test) and Hep3B cells (*p < 0.05 vs. pcDNA‐STAT3 alone treatment, **p < 0.01 vs. pcDNA‐JAK1 alone treatment, mean ± SEM, *t*‐test). (F) Schematic depicts the overview of the mechanism: ATO and 5‐FU/cisplatin synergistically depress LIF/JAK/STAT and NF‐kB pathways

To further validate the mechanism underlying ATO‐induced differentiation in HCC cells, the activation status of the JAK‐STAT signaling pathway was evaluated by western blot analysis. Results showed that pretreatment with ATO significantly decreased the levels of phosphorylated JAK1 and STAT3 in both HCC cell lines. Downregulation of LIF, JAK1, and STAT3 in HCC cells after ATO treatment was also observed (Figure 5A). Since JAK‐STAT signaling was reported to trigger NF‐kB pathway activation, we further explored whether ATO depressed NF‐kB pathway activation by western blot analysis. Five members of the NF‐kB signaling pathway (P50, P52, P65, c‐rel, RELB) were significantly downregulated by ATO treatment (Figure 5B). We transfected HCC cells with plasmids encoding JAK1 and STAT3 to evaluate their levels and phosphorylation status, so as to confirm the effects of JAK‐STAT signaling on ATO‐induced NF‐kB suppression (Figure 5C). We found that reactivation of either JAK1 or STAT3 partially restored ATO‐induced downregulation of P50 and P65 (Figure 5D).

Next, dual‐luciferase assays were conducted to verify the effects of JAK‐STAT3 signaling on NF‐kB activation. Results indicated that expression of JAK1 or STAT3 not only increased the luciferase activity of the NF‐kB pathway without ATO treatment but also partially rescued the inhibitory effects of ATO treatment on NF‐kB activation in Hep3B cells. Similar results were observed in Huh7 cells (Figure 5E). In addition, we applied siRNAs to deplete key molecules in both signal cascades such as *P65* and *STAT3* genes to explore whether the depletion of them would alter the differentiation action of ATO. The knockdown efficiency of siRNAs was verified by RT‐qPCR and western blot analysis (Figure S1A). In plate clone formation assays, ATO could suppress the clone formation of Huh7 cells as previously shown. We found that simply deletion of *P65* or *STAT3* did not alter the actions of ATO. Interestingly, deletion of *P65* and *STAT3* synergistically could reduce the observed actions of ATO. The number of clones was significantly increased (Figure S1B). Similar results were observed in sphere formation assays. The number of spheres decreased from 20.33 ± 2.03 to 3.67 ± 0.88 after ATO treatment (*p *= 0.0017). However, when *P65* and *STAT3* were depleted, the actions of ATO were reduced with the number of spheres increased to 9.00 ± 1.16 (*p *= 0.02) (Figure S1C). Collectively, our data reveal that ATO exerts its effects by deactivating the LIF/JAK/STAT and NF‐kB cascades synergistically (Figure 5F).

### 3.6 Effects of ATO, 5FU/cisplatin, or combinatorial therapy on ROS, SOD, CAT, and GSH activity

Previous microarray analysis also identified that GSH metabolism is a potential target altered by ATO. It is well known that GSH is a major intracellular thiol compound that plays an important role in the cellular defense against oxidative stress in mammals.^26^ So we measured the ROS levels, the activities of antioxidant enzymes such as superoxide dismutase (SOD), catalase (CAT), and GSH content comparing ATO alone, 5‐FU/cisplatin, and their combinatorial therapies. Here, combinatorial treatment with 3 μM ATO and 60 μM cisplatin/5‐FU for 48 h resulted in an increase in ROS levels compared with ATO or 5‐FU/cisplatin treatment alone in Huh7 cells (Figure S2A and B).

The cellular levels of SOD, CAT, and GSH were also significantly decreased when cells were treated with 3 μM ATO. In comparison with the control group, cells treated with ATO showed SOD, CAT, and GSH levels decreased to (35.67 ± 3.09 U/mg protein, 21.33 ± 2.81 U/ mg protein, and 7.00 ± 1.46 μM/mg protein, *p *< 0.05) for 48 h. Similar results were also observed in the combinatorial treatment group. We found 5‐FU/cisplatin single treatment could also decrease SOD levels (from 64.67 ± 2.03 to 55.67 ± 2.33 and 44.67 ± 2.08, *p *< 0.05) (Figure S2C–E).

## 4 DISCUSSION

Current medical management of advanced HCC is modest and cancer chemotherapies are highly toxic and often nonspecific.^5^, ^32^, ^33^ A potentially less toxic approach to treating this common disease employs agents that modify cancer cell differentiation, termed “differentiation therapy.”^13^ This approach is based on the hypothesis that a proportion of tumor cells exhibit reversible defects in differentiation, which upon appropriate treatment could lead to tumor reprogramming and loss of the capacity for self‐renewal and induction of terminal differentiation.^34^ It has been shown that treatment of acute PML cells with ATO leads to their terminal differentiation.^35^ This breakthrough has greatly expanded the number of studies on the clinical application of differentiation therapy with ATO in solid tumors. Zhang et al. reported ATO may induce HCC CSC differentiation, inhibit cancer recurrence after resection, and prolong survival as a result of downregulation of *GLI1* expression.^22^ Another study implied that ATO can inhibit liver CSCs and metastasis through minichromosome maintenance protein (MCM) 7 by suppressing the transcription activity of serum response factor (SRF)/MCM7 complex.^23^

Here, we found that ATO significantly reduced CD133^+^ cells in Hep3B and Huh7 HCC cell lines. CD133^+^ cells have been well recognized as the subpopulation of CSCs in HCC with high clonogenicity, tumorigenicity, and resistance to conventional chemotherapy.^36^, ^37^, ^38^ Meanwhile, the stemness‐related genes *ABCG2*, *SOX2*, *OCT4*, and *NANOG* ^39^ were downregulated after ATO administration. Furthermore, data from the sphere‐forming assay^40^ and clone‐forming assay^41^ indicated that self‐renewal and the tumor‐forming capacity, which are two important characteristics of CSCs, were significantly attenuated after ATO treatment. The activity of ALDH, another marker of both normal and malignant stem and progenitor cells,^42^ was also found to be decreased upon ATO exposure. These results suggest that ATO holds great potential for inducing differentiation of CSCs in HCC. The CD133^+^ CSCs in HCC exhibited preferential expression of stem cell‐related genes and were more resistant to chemotherapeutic agents as a result of the upregulation of *ABCG2*.^25^ This protein functions as a xenobiotic transporter that may play a major role in multi‐drug resistance.^43^ Like ALDH, it serves as a cellular defense mechanism in response to mitoxantrone and anthracycline exposure.^43^ As previously presented, both *ABCG2* and ALDH were downregulated by ATO, which prompted us to investigate whether HCC cell resistance to conventional chemotherapy could be altered by ATO differentiation treatment. In this study, we report that ATO‐induced differentiation of HCC CSCs effectively potentiated the cytotoxic effects of 5‐FU/cisplatin in vitro and in vivo. Notably, we identified this point in vivo using PDTX models, which makes the result more convincing. PDTX are the only models harboring bona fide tumor targets directly from the patient, and hence their use in drug discovery is promising.^44^ Several PDTX studies have proven effective in paralleling human outcomes, exploring drug‐resistance mechanisms, and identifying targets for second‐line treatment.^45^, ^46^, ^47^ These results could also attribute to ROS accumulation and GSH depletion caused by ATO pretreatment. In fact, another reason that CSCs are more resistant to chemotherapies is partly because of their higher GSH content and antioxidant enzyme.^31^, ^48^, ^49^ In the present study, we have witnessed that ATO combined with 5‐FU/cisplatin could generate more ROS in HCC cells. Meanwhile, ATO can also decrease the level of SOD, CAT, and GSH, which could explain why CSC become more sensitive to chemotherapies such as cisplatin after ATO pre‐treatment.^50^ GSH metabolism as a potential target signaling was screened out in the following oligo‐microarray analysis.

In the present study, we explored the possible molecular mechanisms underlying ATO‐induced differentiation using a human whole‐genome oligo‐microarray. The JAK‐STAT signaling pathway was chosen for further investigation. We found that both LIF and JAK1 were downregulated, and JAK1 and STAT3 were dephosphorylated by ATO treatment in a time‐dependent manner. The JAK‐STAT signaling pathway was identified and has been implicated in diverse biological processes, including immune response, hematopoiesis, neurogenesis, oncogenesis, maintenance of pluripotency, and control of many populations of stem cells.^28^ LIF, a member of the IL‐6 cytokine family, binds to its specific receptor and leads to the recruitment and activation of the JAK pathway. STAT3 is then phosphorylated and translocated to the nucleus where it initiates regulation of the transcription of a wide range of genes.^51^ It is believed that overactivation of JAK‐STAT in mammals leads to the initiation of several types of cancers. Studies reported that upregulation of the JAK‐STAT signaling pathway and inhibition of STAT3 decrease the stem cell population in breast cancer.^52^

The interplay of JAK‐STAT and NF‐kB is a common feature in several types of cancer. A previous study illustrated that the crosstalk between JAK1/STAT3 and NF‐kB is central for tumor progression.^53^ In multiple myeloma, suppression of IL‐6‐mediated STAT3 activation and NF‐kB inhibition could inhibit proliferation, induce apoptosis, and increase chemosensitivity.^54^ Moreover, in liver cancer, a recent paper declared that certain lncRNAs could suppress liver CSC expansion via competitively binding the IL‐6 promoter and suppressing IL‐6 transcription to block NF‐kB‐mediated transcription.^55^ It has been further elucidated that constitutively activated STAT3 maintains constitutive NF‐kB activity in cancers by inhibiting its export from the nucleus. The sustained activation of STAT3 and NF‐kB ultimately leads to stimulation of pro‐survival, proliferative, and angiogenesis‐related genes in cancer. In agreement with these previous reports, we explored the crosstalk between the JAK1/STAT3 and NF‐kB pathways in HCC cells with ATO treatment. Upregulation of either JAK1 or STAT3 could partially restore ATO‐induced downregulation of P50 and P65. Promotor analysis indicated that NF‐kB transcription activities were enhanced by JAK1/STAT3 upregulation and, in contrast, were suppressed by ATO. Furthermore, we found single deletion of P65 or STAT3 did not alter the proliferation and self‐renewal inhibitory effect of ATO significantly, but deletion of P65 and STAT3 synergistically could reduce those effects of ATO. These results indicated that interactions existed between JAK1/STAT3 and NF‐kB pathways and possible key molecules existed as action points of ATO in both signaling pathways, which could affect the therapeutic effect of ATO. However, more evidence concerning direct interactions or indirect regulation need to be further elucidated in the future.

In summary, our study has demonstrated that ATO treatment induced differentiation of CSCs in HCC and potentiated the cytotoxic effects of 5‐FU/cisplatin through synergistic inhibition of the LIF/JAK1/STAT3 and NF‐kB signaling pathways. However, whether combinatorial treatment with ATO and 5‐FU/cisplatin can effectively inhibit HCC cell migration and metastasis needs further study. Although a similar approach with ATO has been already applied in HCC as previously mentioned,^22^, ^23^ we implied potential interactions between two signal pathways for the first time and we tend to put more emphasis on its clinical translational significance such as combination strategy with conventional chemotherapies in the present study, since a previous phase II clinical trial concluded single agent ATO is not active against advanced HCC.^56^ Furthermore, in multiple clinical trials, patients receiving an ATO‐based combination therapy have less extrahepatic metastasis and longer survival.^57^, ^58^ Interestingly, in clinical practice, we witnessed a dramatic AFP decrease in end‐stage HCC patients, who were insensitive to conventional chemotherapy and targeted therapy after 3 to 4 courses of ATO pretreatment. This finding makes our work more meaningful. Thus, we hope these findings could shed new light on the clinical strategy for the treatment of HCC and provide potential guidance to improve the treatment outcomes of patients with HCC.

## CONFLICT OF INTEREST

The authors declare that they have no conflict of interest.

## Supporting information




**Supplementary Figure 1. Actions of ATO on clone and sphere formation weakened after both P65 and STAT3 deletion (A**). RT‐qPCR and western blot analysis were applied to verify knockdown efficiency of P65/STAT3 siRNAs. SiRNA2 was chosen for further experiments. **(B)** Photos of plate clone formation assay show the different densities of cell clones under indicated conditions (upper panel); relative clone count under indicated conditions was shown using a histogram (lower panel, * *P* < 0.05, mean ± SEM, *t‐*test). **(C)** Photos of cell sphere formation show **the** number and size of cell spheres under indicated conditions (Magnification: 200×); relative sphere count was also shown in histogram (* *P* < 0.05; **P < 0.01, mean ± SEM, *t‐*test).

**Supplementary Figure 2. Effects of ATO, 5FU/cisplatin, or combinatorial therapy on ROS, SOD, CAT, and GSH activity (A and B)**. Intracellular ROS were measured by an oxidation‐sensitive fluorescent probe 2′,7′‐dichlorofluorescin diacetate (DCFH‐DA).Photos were taken using an upright fluorescence microscope (Magnification: 100×).The intensity of green fluorescence represents the accumulation of ROS (left panel). ROS levels with indicated therapy were detected by flow cytometry (relative DCFH‐DA fluorescence intensity, ATO+5‐FU: 9643±33.53; 5FU:6831±41.28; ATO:6678±37.28; ATO+5‐FU vs. ATO/5‐FU, *P *< 0.05; ATO+Cis:12151±104.3; Cis:9088±110.2; ATO:9213±98.5; ATO+Cis vs. ATO/Cis, *P *< 0.05, *t*‐test, mean ± SEM) (right panel). **(C‐E)** Effects of ATO, 5FU / Cisplatin or combinatorial therapy on SOD, CAT and GSH activities for 48h. Data are shown as means± SEM from three separate experiments. Statistical analysis was performed according to the Student's *t*‐test (data were all compared to the NC group, * *P *< 0.05; ** *P *< 0.01). **Abbreviations**: ATO, arsenic trioxide; HCC, hepatocellular carcinoma; CSC, cancer stem cell; ALDH, aldehyde dehydrogenase; PDTX, patient‐derived tumor xenograft; 5‐FU, 5‐fluorouracil; KEGG, Kyoto Encyclopedia of Genes and Genomes; Cis, cisplatin

Supporting Information
