# Supplementary figures and images for: Arsenic trioxide induces differentiation of cancer stem cells in hepatocellular carcinoma through inhibition of LIF/JAK1/STAT3 and NF‐kB signaling pathways synergistically
Source: Clin Transl Med. 2021 Feb 23;11(2):e335. doi: 10.1002/ctm2.335 (PMC7901720; doi:10.1002/ctm2.335)

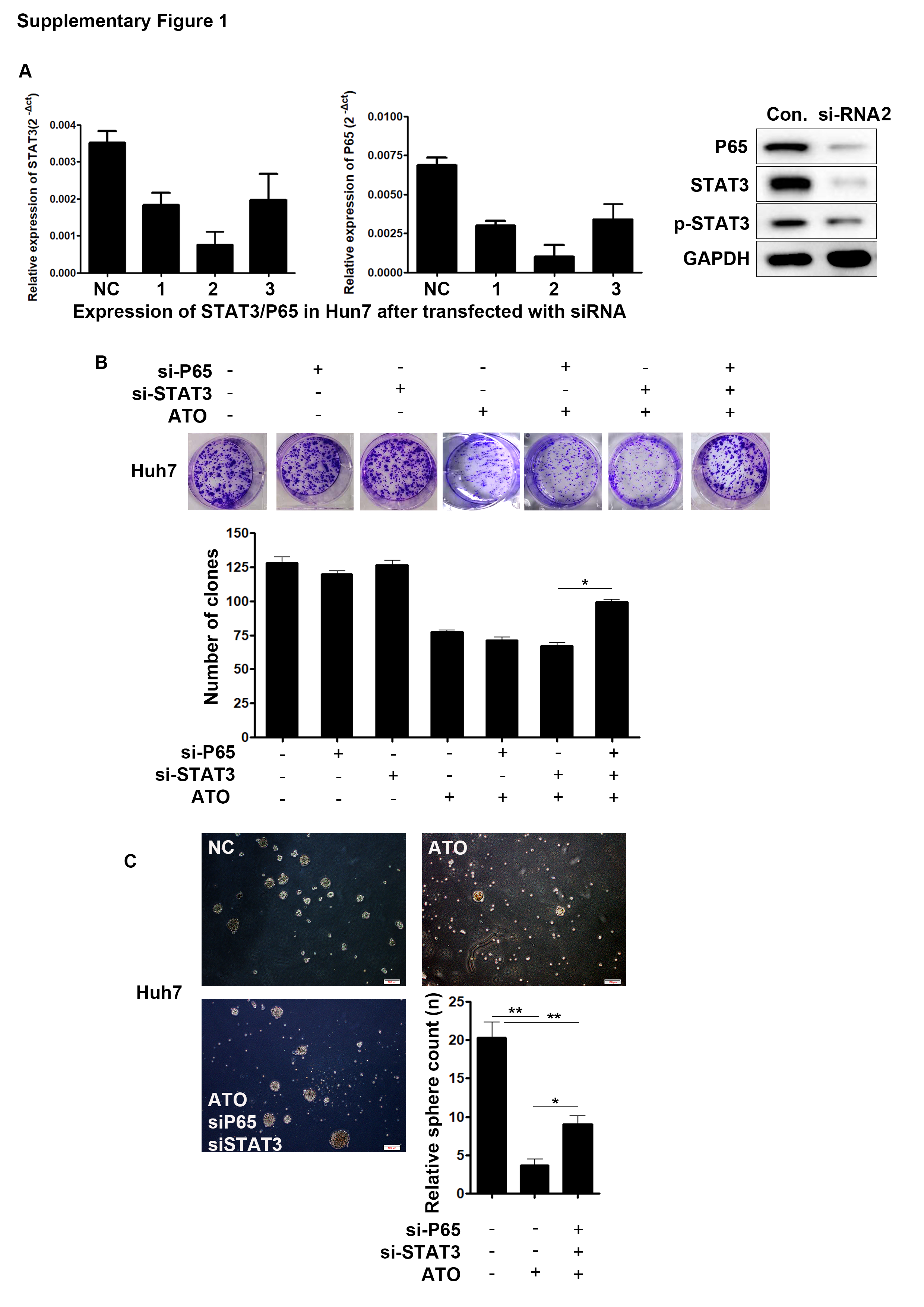

Supplement: Supplementary file 1 — Supplementary Figure 1. Actions of ATO on clone and sphere formation weakened after both P65 and STAT3 deletion (A). RT‐qPCR and western blot analysis were applied to verify knockdown efficiency of P65/STAT3 siRNAs. SiRNA2 was chosen for further experiments. (B) Photos of plate clone formation assay show the different densities of cell clones under indicated conditions (upper panel); relative clone count under indicated conditions was shown using a histogram (lower panel, * P < 0.05, mean ± SEM, t‐test). (C) Photos of cell sphere formation show the number and size of cell spheres under indicated conditions (Magnification: 200×); relative sphere count was also shown in histogram (* P < 0.05; **P < 0.01, mean ± SEM, t‐test). [file CTM2-11-e335-s001.tif]

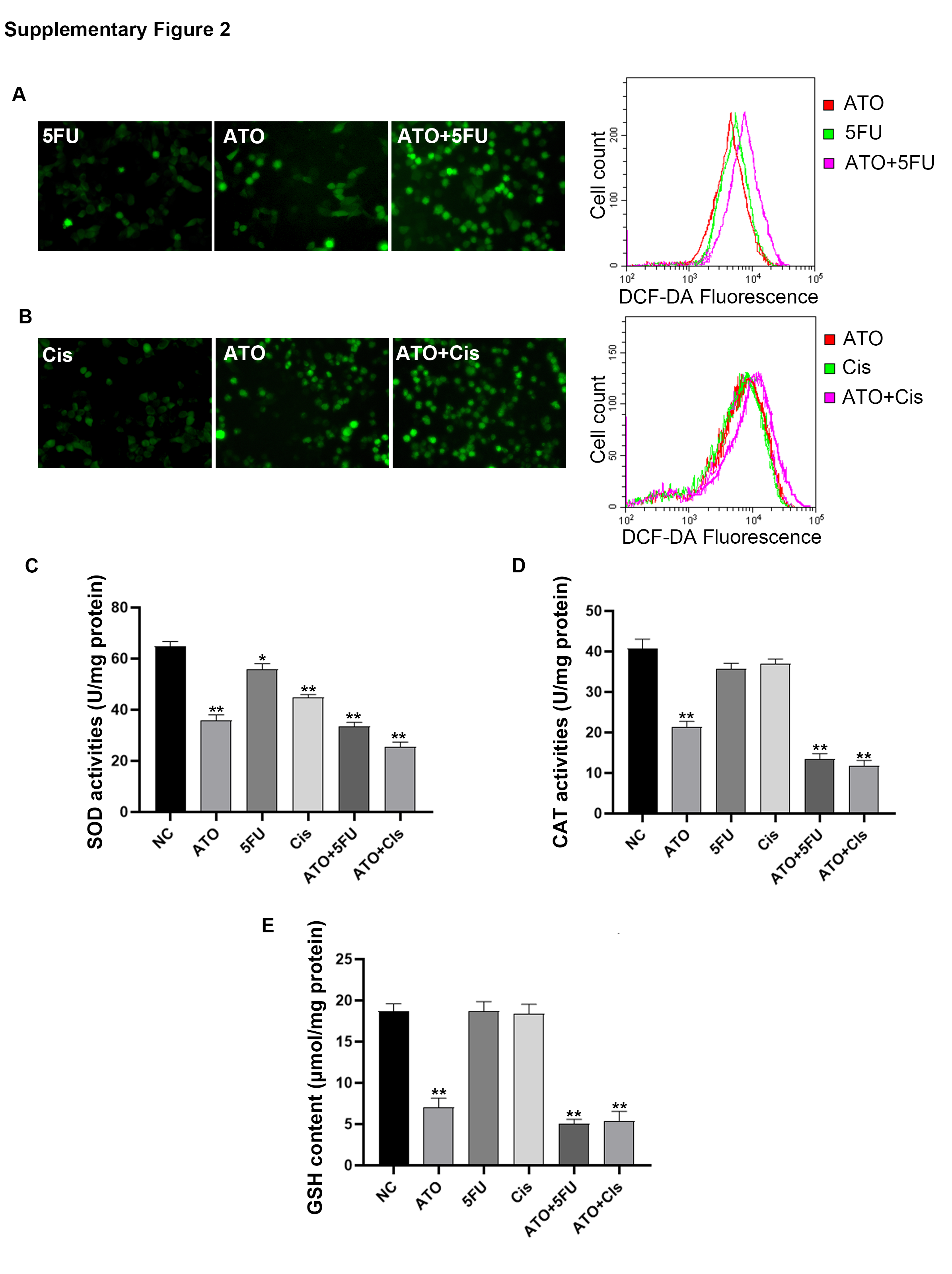

Supplement: Supplementary file 2 — Supplementary Figure 2. Effects of ATO, 5FU/cisplatin, or combinatorial therapy on ROS, SOD, CAT, and GSH activity (A and B). Intracellular ROS were measured by an oxidation‐sensitive fluorescent probe 2′,7′‐dichlorofluorescin diacetate (DCFH‐DA).Photos were taken using an upright fluorescence microscope (Magnification: 100×).The intensity of green fluorescence represents the accumulation of ROS (left panel). ROS levels with indicated therapy were detected by flow cytometry (relative DCFH‐DA fluorescence intensity, ATO+5‐FU: 9643±33.53; 5FU:6831±41.28; ATO:6678±37.28; ATO+5‐FU vs. ATO/5‐FU, P < 0.05; ATO+Cis:12151±104.3; Cis:9088±110.2; ATO:9213±98.5; ATO+Cis vs. ATO/Cis, P < 0.05, t‐test, mean ± SEM) (right panel). (C‐E) Effects of ATO, 5FU / Cisplatin or combinatorial therapy on SOD, CAT and GSH activities for 48h. Data are shown as means± SEM from three separate experiments. Statistical analysis was performed according to the Student's t‐test (data were all compared to the NC group, * P < 0.05; ** P < 0.01). Abbreviations: ATO, arsenic trioxide; HCC, hepatocellular carcinoma; CSC, cancer stem cell; ALDH, aldehyde dehydrogenase; PDTX, patient‐derived tumor xenograft; 5‐FU, 5‐fluorouracil; KEGG, Kyoto Encyclopedia of Genes and Genomes; Cis, cisplatin [file CTM2-11-e335-s002.tif]
