# Supplementary material for: Arsenic trioxide induces differentiation of cancer stem cells in hepatocellular carcinoma through inhibition of LIF/JAK1/STAT3 and NF‐kB signaling pathways synergistically
Source: Clin Transl Med. 2021 Feb 23;11(2):e335. doi: 10.1002/ctm2.335 (PMC7901720; doi:10.1002/ctm2.335)
Supplement: Supplementary file 3 — Supporting Information [file CTM2-11-e335-s003.docx]

| Genes | Sequence |
| --- | --- |
| Sox2 | F:5’-aaatgggaggggtgcaaaagaggag-3’  R:5’-cagctgtcatttgctgtgggtgatg-3’ |
| ABCG2 | F:5’-tcatcagcctcgatattccatct-3’  R:5’-ggcccgtggaacataagtctt-3’ |
| CD133 | F:5’-tggatgcagaacttgacaacgt-3’  R:5’-atacctgctacgacagtcgtggt-3’ |
| Notch1 | F:5’-cctgagggcttcaaagtgtc-3’  R:5’-cggaacttcttggtctccag-3’ |
| Nanog | F:5’-aatacctcagcctccagcagatg-3’  R:5’-tgcgtcacaccattgctattcttc-3’ |
| Oct4 | F:5’-cttgctgcagaagtgggtggaggaa-3’  R:5’-ctgcagtgtgggtttcgggca-3’ |
| JAK1 | F:5’-atttgggttctcggcaatac-3’  R:5’-ggtcatccttgacggtgtaa-3’ |
| LIF | F:5’-gatgttcctgccttagagtcatc-3’  R:5’-cccacagggtacattcatca-3’ |

**Supplementary Table** **S1. Primers used in qPCR assays.**

**Supplementary Table** **S2. siRNA design for P65 and STAT3**

| Genes | siRNAs |
| --- | --- |
| STAT3 | 1.5’-CCGGCTCAGAGGATCCCGGAAATTTCTCGAGAAATTTCCGGGATCCTCTGAGTTTTTG-3’ |
|  | 2.5’-CCGGGGCGTCCAGTTCACTACTAAACTCGAGTTTAGTAGTGAACTGGACGCCTTTTTG-3’ |
|  | 3.5’-CCGGCATCTGAAACTACTAACTTTGCTCGAGCAAAGTTAGTAGTTTCAGATGTTTTTG-3’ |
|  |  |
| P65 | 1.5’-CCGGCCCTCAGCACCATCAACTTTGCTCGAGCAAAGTTGATGGTGCTGAGGGTTTTTG-3’ |
|  | 2.5’-CCGGAGAAGACATTGAGGTGTATTTCTCGAGAAATACACCTCAATGTCTTCTTTTTTG-3’ |
|  | 3.5’-CCGGAGGCCATATAGCCTTACTATCCTCGAGGATAGTAAGGCTATATGGCCTTTTTTG-3’ |

**Supplementary Table S3.** The enrichment score (ES) and normalized enrichment score (NES) for analyzed pathways.

| Pathways | ES | NES | Nominal *P* value |
| --- | --- | --- | --- |
| Jak-STAT signaling pathway | -0.38 | -1.56 | 0.003 |
| P53 signaling pathway | 0.52 | 1.87 | 0.0001 |
| Metabolism of xenobiotics by cytochrome P450 | 0.46 | 1.66 | 0.004 |
| PPAR signaling pathway | -0.34 | -1.27 | 0.102 |
| Tryptophan metabolism | 0.49 | 1.54 | 0.013 |
| ECM-receptor interaction | 0.37 | 1.40 | 0.046 |
| Valine, leucine and isoleucine degration | -0.26 | -0.58 | 0.947 |
| Glutathione metabolism | 0.57 | 1.97 | 0.001 |
